# Identification of a serum-based microRNA signature that detects recurrent oral squamous cell carcinoma before it is clinically evident

**DOI:** 10.1038/s41416-023-02405-9

**Published:** 2023-10-05

**Authors:** Rebecca Towle, Christopher T. D. Dickman, Sara A. MacLellan, Jiahua Chen, Eitan Prisman, Martial Guillaud, Cathie Garnis

**Affiliations:** 1 Department of Integrative Oncology, British Columbia Cancer Research Centre, Vancouver, BC Canada; 2 Department of Statistics, University of British Columbia, Vancouver, BC Canada; 3 Division of Otolaryngology, Department of Surgery, University of British Columbia, Vancouver, BC Canada

**Keywords:** Predictive markers, Oral cancer detection

## Abstract

**Background:**

Survival rates for oral squamous cell carcinoma (OSCC) have remained poor for decades, a fact largely attributable to late-stage diagnoses and high recurrence rates. We report analysis of serum miRNA expression in samples from patients with high-risk oral lesions (HRL, including OSCC/carcinoma in situ lesions) and healthy non-cancer controls, with the aim of non-invasively detecting primary or recurrent disease before it is clinically evident.

**Methods:**

Discovery, test, and validation sets were defined from a total of 468 serum samples (305 HRL and 163 control samples). Samples were analysed using multiple qRT-PCR platforms.

**Results:**

A two-miRNA classifier comprised of miR-125b-5p and miR-342-3p was defined following discovery and test analyses. Analysis in an independent validation cohort reported sensitivity and specificity of ~74% for this classifier. Significantly, when this classifier was applied to serial serum samples taken from patients both before treatment and during post-treatment surveillance, it identified recurrence an average of 15 months prior to clinical presentation.

**Conclusions:**

These results indicate this serum miRNA classifier is effective as a simple, non-invasive monitoring tool for earlier detection of recurrent disease when lesions are typically smaller and amenable to a wider array of treatment options to improve survival.

## Background

Oral squamous cell carcinoma (OSCC) is the most commonly diagnosed form of head and neck cancer and the eighth most commonly diagnosed cancer type worldwide [1]. Survival rates for oral cancer are poor, driven by frequent late-stage diagnoses and high recurrence rates. The 5-year recurrence rate for oral cancer is ~50%, with most cases recurring in the first two years after treatment. Current post-treatment standard of care is ‘watchful waiting’; however, detecting recurrent disease by clinical exam alone is challenging, often relying on presentation of new symptoms (bleeding, pain, etc.). Thus, ~90% of recurrent cases have progressed to metastasis at the time of diagnosis (leaving limited treatment options). As a result, the 5-year overall survival rate for patients with recurrent oral tumours is only ~30%. Earlier detection of recurrent lesions and/or primary lesions – when they are smaller and have yet to spread – is advantageous from a treatment perspective, as surgery and radiation treatments may still be feasible. In practice, tools that facilitate earlier detection of disease will improve on visual inspection by triggering earlier evaluation by confirmatory imaging (e.g., CT or PET) and facilitating expanded use of treatments known to improve disease outcomes.

Advances in RNA detection sensitivity have led to the discovery of extracellular RNA species in bodily fluids, which may have utility as biomarkers for delineating disease states. This includes mRNA, ribosomal RNA, long non-coding RNA, exonic circular RNA, and small non-coding RNA molecules like miRNA [2]. The majority of extracellular RNA species are detected in vesicles like exosomes, nano- and micro-vesicles, or apoptotic bodies. Multiple reports have described the isolation of extracellular miRNAs from body fluids such as saliva, serum, plasma, urine, breast milk, and CSF [3–6]. It is believed that the extracellular vesicle protects the RNA cargo from degradation and therefore lends itself as a good source for biomarker discovery. For oncology, circulating tumour-specific miRNAs have been identified for numerous cancer types, including head and neck cancer, and are believed to have utility as non-invasive biomarkers [7–9]. MiRNAs are well known for their role in cell growth and proliferation as they regulate several biological processes related to cancer development [10, 11]. They are often deregulated in tumour cells themselves, as well as within extracellular vesicles that the tumour cells release into circulation [12]. There are several reports analysing miRNA expression in serum or plasma of individuals with oral cancer, although these reports are often of limited utility due to small sample sizes, biased miRNA candidate selection, or lack of proper validation [13–18].

Herein, we report on the discovery and validation of a miRNA classifier that is capable of discriminating serum from patients with early-stage oral squamous cell carcinoma (OSCC) and/or carcinoma in situ (CIS) from demographically matched non-cancer control patients with high sensitivity and specificity. Significantly, this miRNA classifier was also able to identify recurrent OSCC before it was clinically evident.

## Methods

### Sample acquisition

Initial blood samples used for discovery and validation were collected from patients with either CIS or OSCC (collectively termed high-risk lesions [HRLs]). Control serum samples from individuals without cancer were demographically matched for sex, age, and smoking history. Control samples were obtained from a pan-Canadian study on the early detection of lung cancer [19]. Serial samples were collected from patients treated by head and neck surgeons at Vancouver General Hospital. Consent was obtained from all patients prior to serum collection and use of human specimens in this work was approved by the University of British Columbia ethics board (H14-00349). Demographic information is listed in Supplemental Table 1.

All blood samples were collected in SST vacutainer tubes then allowed to clot for 30 min at room temperature. Samples were then centrifuged at room temperature for 15 min at 1500 rcf, with serum then collected and frozen in aliquots at −80 °C within 2 hours of collection.

### RNA extraction

RNA was extracted from 200 μl of serum using the miRNeasy Mini Kit (Qiagen) as previously described [18].

### qRT-PCR

Initial training cohort (51 non-cancer, 48 HRL) qRT-PCR analysis was performed using the miRCURY LNA Universal RT miRNA PCR Human Panel I and II (Exiqon) (742 miRNAs). Generation of cDNA was completed using a miRCURY LNA Universal cDNA synthesis kit on 19.2 μl of serum RNA, with cDNA then quantified using SYBR Green master mix according to manufacturer recommendations. All assays were examined for distinct melting curves, and samples with multiple Tm or CT > 35 were excluded from analysis.

TaqMan qRT-PCR was performed on selected candidate miRNAs using either custom pre-spotted plates or individual TaqMan assays with each assay run in triplicate. RNA extracted from serum was quantified using a Qubit 3.0 (Thermo Fisher) using the RNA HS reagents. The list of TaqMan miRNA primers and assay IDs is available in Supplemental Table 2. Reverse transcription was performed on 300 ng of RNA using TaqMan miRNA reverse transcription kits. A 12-cycle preamplification step was performed using the TaqMan PreAmp master mix. For qPCR, Universal Master Mix II was used according to manufacturer recommendations.

### Data analysis

For SYBR qRT-PCR, miRNAs were excluded from analysis if they were not expressed with a CT < 35 in at least half of the control group or half of the disease group. We excluded 162 miRNAs present on Human Panel I and II that had been shown to have altered detection dependent on sample haemolysis [20].

SYBR and TaqMan analysis expression was normalised to an endogenous control miR-23b-3p chosen based upon its value using the geNorm algorithm showing it was the least variable across samples [21]. CT values for miRNAs that were not detected were set to the threshold of detection (35 for SYBR and 37 for TaqMan). To normalise the CT of potential candidate miRNAs, the CT value was subtracted from the CT of the chosen normalising miRNA (ΔCT). The ΔCT data was linearised using the formula CT_linear_ = 2^(−ΔCT normalised)^.

The normality of data was determined using the Shapiro-Wilk test and equality of variance using a F-test. A two-tailed Mann-Whitney U test was used to determine significance between two groups. Significance within more than two groups was determined by the Kruskal-Wallis test. The appropriate sample size for our initial training set was calculated to be 49 HRL and 49 control based on a recurrence prevalence of 50%, power of 80%, and alpha of 5% [22].

## Results

### Biomarker training analysis

#### SYBR qRT-PCR analysis

**Fig. 1:**
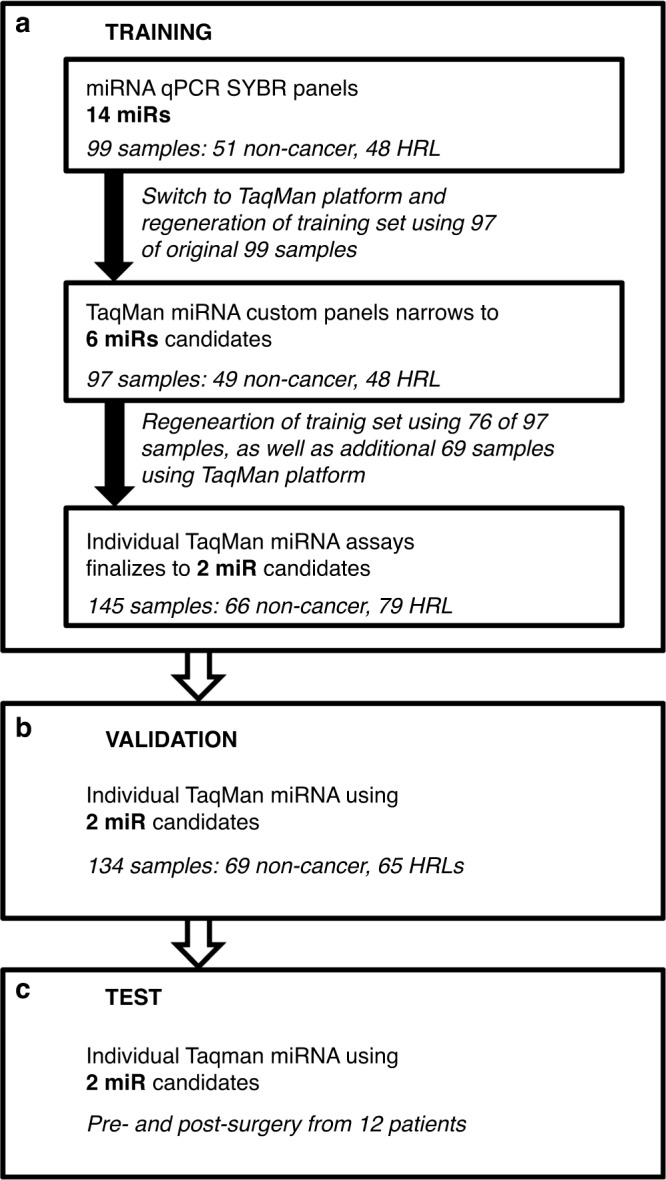
Flow chart describing study design. **a** Determination of classifier first with SYBR LNA technology and then with TaqMan qPCR identifying a final 2 miRNA classifier panel. This panel was subsequently validated using an independent sample set (**b**) and tested on a panel of 12 paired Pre- and Post-Surgery samples (**c**).

The study design and an overview of the sample distribution is illustrated in Fig. 1. We initially analysed miRNA expression in 99 serum samples, including samples from 48 individuals with HRLs and 51 control cases using the Ready-to-Use PCR, Human panel I and panel II with the miRCURY LNA Universal RT miRNA PCR. Of the 742 miRNAs that were queried by qRT-PCR, 162 were known to be affected by haemolysis and were excluded from further analysis [20]. Of the remaining miRNAs, 415 were detected in at least one serum samples with only 14 miRNAs being detected in every sample. We further limited analysis to the miRNAs which were expressed in ≥50% of the HRL group or ≥50% of the control group. After this selection, 106 miRNAs remained.

**Fig. 2:**
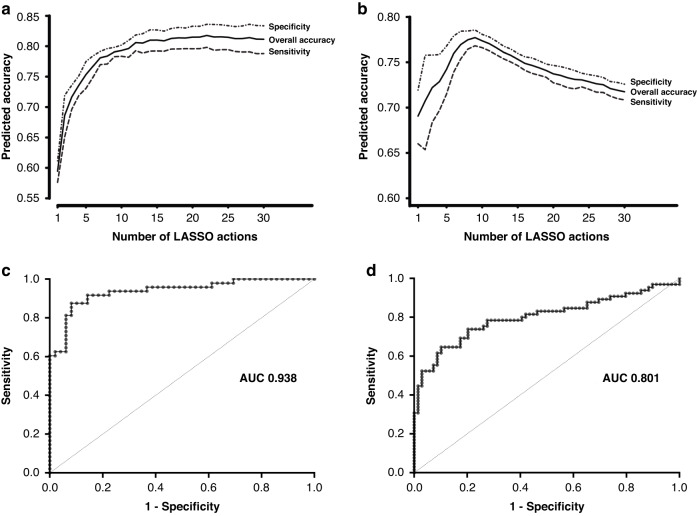
Average accuracy for preliminary SYBR and TaqMan analysis. The average sensitivity, specificity and overall accuracy in determining oral cancer status per LASSO action while examining randomly partitioned validation sets of miRNA expression in the (**a**) SYBR and (**b**) TaqMan training sets. Resampling was performed 10,000 times and therefore the averages shown are based on models with the inclusion of different miRNAs under examination. (**c**) ROC illustrating the 6 miRNA classifier on the TaqMan training set. (**d**) Performance of the 2 miRNA classifier in the validation sample set (69 non-cancer and 65 high-risk lesion [HRL] samples).

We performed a LASSO analysis on the qRT-PCR data for the remaining 106 miRNAs to select those that would best discriminate between the two groups (HRL and non-cancer cases). This analysis selected 16 miRNAs as the best classifiers (Fig. 2a). Using a random forest analysis this classifier was determined to classify the samples with 70.6% sensitivity and 82% specificity. The miRNAs selected and the order they were included in or excluded from the model is shown in Supplemental Table 3.

### TaqMan qRT-PCR Analysis

To ensure that the classifier we identified was not skewed by potential LNA primer artefact, we evaluated the same training set samples (two control samples were excluded from this second analysis due to insufficient input RNA) using the TaqMan platform, analysing the top 14 miRNAs comprising the classifier defined by the SYBR LASSO analysis. LASSO and bootstrap analyses were repeated (Fig. 2b). The top 14 miRNAs from the SYBR analysis were re-evaluated. Two were eliminated based on the LASSO action accuracy plot (Fig. 2a) where the input of each additional action (miRNA) into the classifier is minimal after 13 lasso actions. Additionally, analysis of these two miRNAs individually revealed neither was statistically significant between the control and tumour samples.

Using the TaqMan system the model reached peak accuracy with nine LASSO actions (six miRNAs [Supplemental Table 4]). A comparison between selected miRNAs from the training sets for both SYBR and TaqMan Lasso analyses (Supplemental Tables 3 and 4) shows that the six most relevant miRNAs were consistent between groups despite having different primer designs and amplification detection systems. Analysis for each miRNA for both methods is presented in Supp. Figs. 1 and 2. The ROC analysis using TaqMan data for the six miRNA candidates resulted in an AUC of 0.938 and sensitivity and specificity of 91.7% and 85.7%, respectively (Fig. 2c).

**Fig. 3:**
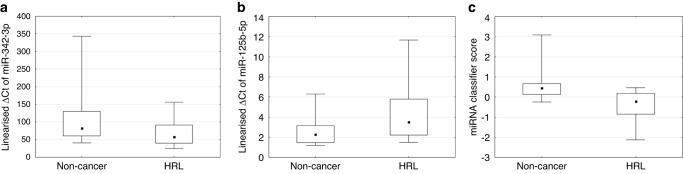
QPCR results for each miRNA and miRNA classifier score. Linearised ΔCT values of (**a**) miR-342-3p alone (**b**) miR-125b-5p alone, and (**c**) combined miR-342-3p and miR-125b-5p in non-cancer (*n* = 66) and HRL (*n* = 79) serum samples. ΔCT= −ΔCT = Ct(reference miR-23b-5p)–Ct(miR of interest).

To evaluate the contribution of each of the six candidate miRNAs, we performed a forward stepwise linear discriminate analysis (LDA) using the TaqMan training set (*n* = 145) (Fig. 1a). This analysis revealed miR-125b-5p gives the strongest performance (65% correct classification), with a moderate increase in performance with the addition of miR-342-3p (Fig. 3). The impact on the classifier by adding the remaining miRNA candidates was negligible.

### Biomarker validation analysis

Expression analysis of this two-miRNA classifier in an independent validation set of serum samples was completed using TaqMan qRT-PCR. This set included 65 HRL samples and 69 control samples. The validation ROC resulted in an AUC of 0.801, a modest decrease from the training set (Fig. 2d). Both sensitivity and specificity were calculated to be 74% for the independent validation set.

### Defining a threshold

**Fig. 4:**
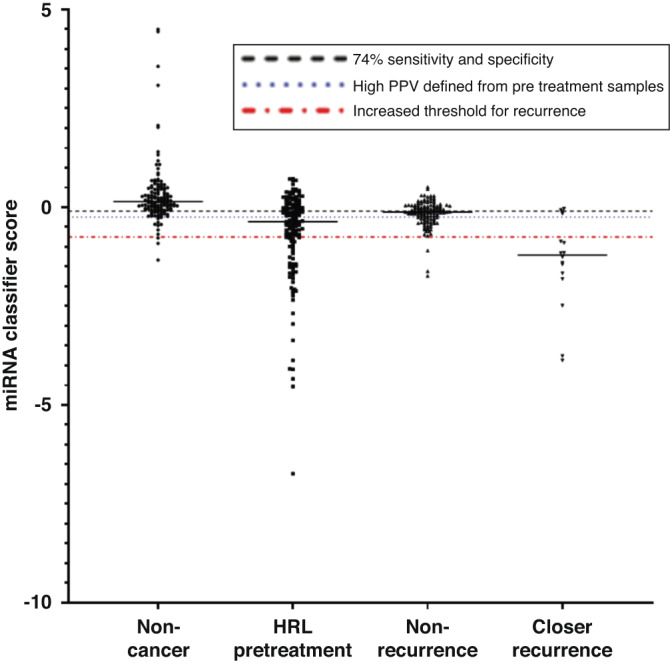
MiRNA classifier score comparing non-cancer (*n* = 135), pre-treatment HRLs (*n* = 144), post-treatment HRLs with no recurrence >2 years after initial treatment (*n* = 121), and serial post-treatment serum samples collected close to recurrence date (average 15 months before recurrence treatment, *n* = 16). Dotted lines indicate potential thresholds for determining recurrence. The Centre line indicates the median.

For this test to have clinical utility, a threshold for determining a positive or negative result for an individual test must be defined. There are several approaches for identifying a threshold or cut point. However, the threshold ultimately has to make sense clinically. We selected the threshold to have the highest positive predictive value (PPV = 89%, Fig. 4). While creating a conservative threshold with a high PPV will result in some false negative results for which the patient would continue with the current standard of care (i.e., clinical exam), we can be quite certain that the positive biomarker results are indicative of the presence of disease. This would limit hesitation to undertake further investigative tests such as PET CT or MRI and limit any undue stress to the patients caused by false positives.

### Effect of treatment on biomarker results

**Fig. 5:**
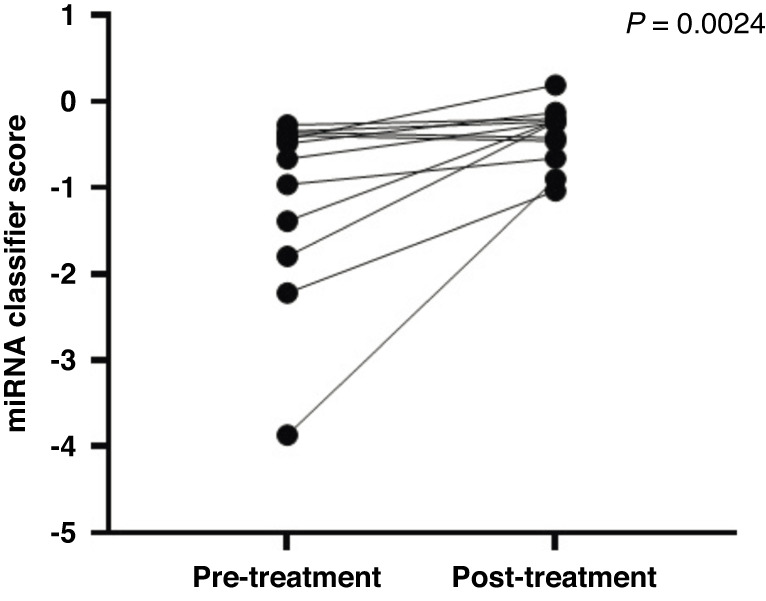
Classifier performance comparing pre-treatment and post-treatment serum samples. Post-surgery samples (*n* = 12) were taken with a mean of 145 days after surgical removal of lesions. Only patients with clear surgical margins were included. A Wilcoxon signed-rank test was used to determine significance.

To demonstrate that the classifier is specific to the presence of the tumour, we analysed 12 samples that were obtained at the time of diagnosis and post-treatment (an average of 145 days post-treatment). Samples were included in this analysis if (1) the pre-treatment serum was positive for the classifier (i.e., the classifier indicated the presence of disease) and (2) surgical margins were free of severe dysplasia or any higher grade lesion. The majority (83%) of post-treatment samples showed an altered classifier score trending towards normal (Fig. 5).

### Serial assessment of the miRNA classifier during post-treatment follow-up

**Fig. 6:**
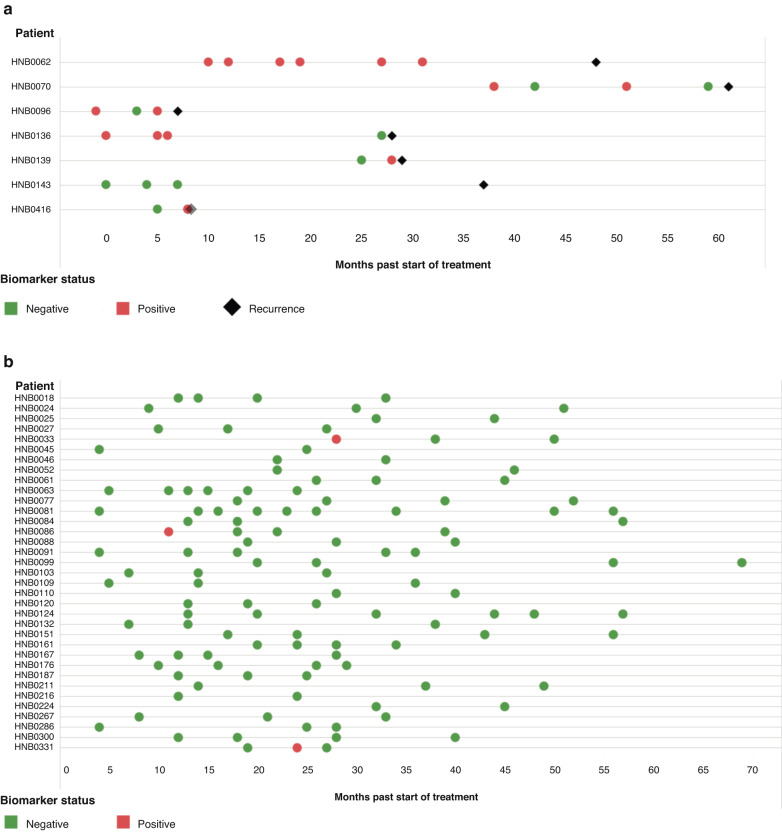
Status of classifier over time in patients with OSCC. Classifier results from serial serum samples collected from follow-up appointments of patients diagnosed with OSCC who (**a**) have recurrence and (**b**) have not exhibited recurrence. Green denotes that the classifier indicated no recurrent disease and red indicates classifier result indicated the presence of recurrent disease. The time point at which recurrence was detected (by imaging or biopsy) is indicated with a black diamond.

To assess the potential utility of the classifier as a tool for post-treatment surveillance, we obtained serial blood samples from 42 patients. Patients were followed for a minimum of two years with an average of 36.5 months where samples were collected at each post-treatment follow-up appointment for a total of 145 samples. Of the 42 cases, seven had recurrent disease. Using the threshold defined via the validation and test sets, the sensitivity and specificity for this group were 75% and 62.5% respectively. However, adjusting the threshold vastly improved the sensitivity to 92% and specificity remained at 75% (Fig. 6). We asked whether treatment type had an impact on either the individual normalised Ct values for each of the miRNAs or the classifier score. No differences were detected (Supplemental Fig. 3). The average time for recurrence detected clinically was 31 months with the minimum being seven months and the maximum at 61 months. The classifier was able to detect the recurrent disease in all cases except one (Fig. 6a). However, we were only able to obtain blood samples for this case 30 months before clinical recurrence; therefore, the recurrent disease may not have been present at that time. The classifier detected recurrence on average 15 months post-treatment, with a minimum of five months and a maximum of 38 months. In addition, the classifier was able to detect recurrent disease an average of 17 months earlier than the clinical exam (Fig. 6a). The remaining 35 patients that were followed were recurrence-free by clinical exam at the last date examined by a physician (Fig. 6b). The false positive rate for the non-recurrent samples was ~2%, assuming the patients remain recurrence-free.

## Discussion

In this report, we describe a robust serum miRNA expression classifier capable of delineating recurrence in OSCC before it is clinically evident. The lack of reproducibility in previous studies seeking to identify serum or plasma miRNA biomarkers for oral cancer detection may be attributable to multiple factors [13–17]. One is possible bias in classification due to the use of a small training set. Another driver of unreproducible results may be limiting the analysis to a small set of pre-selected miRNAs. Multiple groups have selected miRNAs based on deregulation observed in OSCC tissues [16, 17]; however, it is now known that miRNAs released by tumour cells do not always reflect the miRNA signature from the donor cell or tissue [23]. Ignoring the impact of haemolysis on serum miRNA expression can also drive a lack of reproducibility since the rupture of red blood cells and subsequent release of their contents (including miRNAs) into the surrounding fluid (e.g., blood plasma or serum) can impact a blood-based biomarker [20]. Significantly, small RNA candidates defined as salient for oral cancer detection in other instances have included miRNAs that are known to be impacted by haemolysis [20]. Finally, the absence of an independent sample set for validation can limit reproducibility.

In addition to addressing the above confounding factors in the design for the work reported here, we also used two different qRT-PCR systems as a technical confirmation (a locked nucleic acid SYBR-based system and TaqMan). These systems use two different primer designs, thus ensuring that the miRNA values we obtained were not PCR artefacts. As miRNAs are generally only ~22 nucleotides in length, it can be difficult to generate primers that generate reproducible PCR data. We observed this in our data with miR-346, which we initially found to be more highly expressed in cancer-associated serum samples as compared to controls (where it was mostly undetected) using the SYBR-based qRT-PCR system. However, with the use of TaqMan primers, miR-346 was undetectable (>35 Cts) in both cancer and non-cancer serum samples. Because of this unreliability across platforms, we excluded miR-346 from further analysis.

There are several factors that must be considered when analysing the performance of a biomarker and deciding on the correct threshold to be used clinically. AUC, sensitivity, and specificity values are useful for the evaluation of a marker; however, they do not specify “optimal” cut points directly. There are several approaches that use both sensitivity and specificity for threshold selection [24–29]. Ultimately, the threshold selected must be clinically useful. In this vein then, there are two main points to consider for an oral cancer biomarker. The first is how well the biomarker performs compared to the current gold standard (clinical exam/watchful waiting) and the second is what is the consequence of a positive test. In this case, it would result in additional imaging. The riskier the consequence (i.e., invasive surgery vs. imaging) the higher the required specificity. In our case, we selected a threshold with higher sensitivity and with lower specificity as we wanted to limit the number of false positives (and the number of people receiving additional, unnecessary imaging) as much as possible. While this results in increased false negatives, these would occur no more often than the current gold standard of care. This could explain why in some of the serial recurrence cases (Fig. 6a) we observe a positive and then a negative test. However, what is striking is that the recurrence cases often (71% of cases) have multiple positive cases over time whereas the non-recurrent cases do not. This could potentially be useful in the clinical interpretation of the test in the future. As we accrue additional samples and follow patients over longer periods of time, we will be able to hone this threshold to give more robust results.

The miRNA classifier identified in this work includes miRNAs that have been individually implicated in malignant processes in the past. The miR-125 family of miRNAs, consisting of three homologous members (miR-125a, miR-125b-1, and miR-125b), are involved in a wide variety of cellular processes including cell differentiation, proliferation, metastasis, apoptosis, drug resistance, and tumour immunity [30]. MiR-125b has been variably reported as up-regulated in several cancer types and down-regulated in several other cancer types [31]. This functioning of miR-125b as either an oncogene or tumour suppressor is dependent on different molecular contexts and tumour microenvironments. In OSCC, miR-125b has been reported as down-regulated and functioning as a tumour suppressor [32–34]. This down-regulation in OSCC is also associated with poor prognosis [35]. Interestingly, we observed an up-regulation of miR-125b in serum samples from HRL patients as compared with samples from non-cancer control subjects. Circulating miRNAs are released from all cell types and vesicles originating from tumour cells have been reported to have a miRNA signature that is not always reflective of the miRNA expression pattern observed within the tumour cells from which they originated [23]. Further, some miRNAs may be selectively packaged into extracellular vesicles and function as cell-cell communicators that promote tumorigenesis [23, 36, 37]. Similarly, miR-342 has been reported to function as a tumour suppressor in various cancer types, playing a role in proliferation, migration, apoptosis, metabolism, and drug resistance [38–44]. In oral cancer, miR-342 has been reported as being down-regulated, functioning as a tumour suppressor by targeting of LASP1 [45]. In our analysis, miR-342-3p was observed to be down-regulated in serum samples from oral cancer patients as compared to serum samples from non-cancer controls. It is possible in this instance that a marked absence of miR-342-3p expression in tumour cells precluded packaging of this miRNA into vesicles for release into the blood, thus accounting for its reduced expression.

The dire need for new tools to help manage follow-up for post-treatment oral cancer is driving the investigation of circulating tumour cells, HPV blood markers, and saliva proteomics and methylomics [46–50]. However, results are preliminary for these markers and none of these findings are currently available as commercial tools. Earlier detection of recurrent lesions and/or primary lesions –when they are smaller and have yet to spread – is advantageous from a treatment perspective, as surgery and, in some cases, radiation treatments are still feasible. These treatments have the potential to increase survival rates by 20% [51, 52]. The current standard for post-treatment follow-up – clinic visits with a physical exam every 3–6 months for several years – has limited efficacy for detecting early recurrence [53]. Post-treatment imaging by PET or PET/CT following baseline assessment (within six months) has a high sensitivity for detecting recurrence (0.95 and 0.91, respectively) compared to conventional CT (0.67) [54–56]. However, all current imaging methods are prone to false positives, require radiation exposure, and are often inaccessible/costly. Therefore, they are not routinely used for long-term follow-up [51, 57]. The classifier for oral cancer recurrence we have described herein will significantly improve on visual inspection/ physical exam. It will do this by facilitating more stringent follow-up for select patients and the strategic use of confirmatory imaging at earlier time points, setting the stage for wider use of treatments known to improve oral cancer patient outcomes.

High rates of disease recurrence are a key barrier to improving oral cancer survival. Currently, recurrence is only detected by clinical exam as prescribed by ‘watchful waiting’, which often misses the earliest stages of recurrent disease. We have developed a simple, non-invasive test that detects disease recurrence before it is clinically evident. This test, upon further validation, will have significant positive impacts on disease management, facilitating earlier, more aggressive monitoring as well as more effective surgical and/or radiation treatment of recurrence.

### Supplementary information

Supplemental Tables

Supplemental Figures

## Data Availability

Analysed and raw data is available upon request.
