# Supplementary material for: Identification of a serum-based microRNA signature that detects recurrent oral squamous cell carcinoma before it is clinically evident
Source: Br J Cancer. 2023 Oct 5;129(11):1810–7. doi: 10.1038/s41416-023-02405-9 (PMC10667517; doi:10.1038/s41416-023-02405-9)
Supplement: Supplementary file 1 — Supplemental Tables [file 41416_2023_2405_MOESM1_ESM.doc]

**Table 1. Demographics of HRL (high risk lesions) and non-cancer samples in each data set.**

*Supplemental Table 1A. Demographic overview of the training and validation sets.*

|  | **TRAINING SET** | | | | | | **VALIDATION SET** | |
| --- | --- | --- | --- | --- | --- | --- | --- | --- |
|  | **SYBR** | | **TAQMAN (6 miRNA)** | | **TAQMAN (2 miRNA)** | |  |  |
|  | **HRL** | **NON-CANCER** | **HRL** | **NON-CANCER** | **HRL** | **NON-CANCER** | **HRL** | **NON-CANCER** |
| **Total Patients** | 48 | 51 | 48 | 49 | 79 | 66 | 65 | 69 |
| **Severe Dysplasia** | 0 | NA | 0 | NA | 5 (6.3%) | NA | 0 | NA |
| **CIS** | 18 (37.5%) | NA | 18  (37.5%) | NA | 27 (34.2%) | NA | 1 (1.5%) | NA |
| **SCC** | 30 (62.5%) | NA | 30  (62.5%) | NA | 47 (59.5%) | NA | 64 (98.5%) | NA |
| **Age Mean** | 62 | 63 | 62 | 63 | 60 | 62 | 65 | 63 |
| **Age Range** | 35-93 | 50-75 | 35-93 | 50-75 | 22-92 | 50-75 | 27-92 | 55-78 |
| **Males** | 35 (72.9%) | 29  (56.9%) | 35  (72.9%) | 29 (59.2%) | 53 (67.1%) | 40 (60.6%) | 29 (44.6%) | 37 (53.6%) |
| **Females** | 13 (27.1%) | 22  (43.1%) | 13 (27.1%) | 20 (40.8%) | 26 (32.9%) | 26 (39.4%) | 36 (55.4%) | 32 (46.4%) |
| **Former Smokers** | 24 (50%) | 30  (58.8%) | 24  (50%) | 30 (61.2%) | 24 (30.4%) | 33 (50%) | 19 (29.2%) | 37 (53.6%) |
| **Current Smokers** | 14 (29.2%) | 21  (41.2%) | 14  (29.2%) | 19 (38.8%) | 47 (59.5%) | 33 (50%) | 7 (10.8%) | 32 (46.4%) |
| **Never Smokers** | 10 (20.8%) | 0 | 10  (20.8%) | 0 | 8 (10.1%) | 0 | 39 (60.0%) | 0 |

*Supplemental Table 1B. Demographic overview of pre/post t*reatment patient samples.

|  | **Pre/Post Treatment Set** |
| --- | --- |
| **Total Patients** | 12 |
| **CIS** | 0 |
| **T1** | 7 (58.3%) |
| **T2** | 4 (33.3%) |
| **T3** | 1 (8.3%) |
| **Age Mean** | 67.75 |
| **Age Range** | 45-85 |
| **Males** | 5 (41.7%) |
| **Females** | 7 (58.3%) |
| **Former Smokers** | 4 (33.3%) |
| **Current Smokers** | 3 (25%) |
| **Never Smokers** | 5 (41.7%) |

*Supplemental Table 1C. Demographic overview of recurrent patient* samples.

|  | **Recurrent Patients** |
| --- | --- |
| **Total Patients** | 7 |
| **Age Mean** | 58 |
| **Age Range** | 35-70 |
| **Males** | 4 (57.1%) |
| **Females** | 3 (42.9%) |
| **Former Smokers** | 6 (85.7%) |
| **Current Smokers** | 0 |
| **Never Smokers** | 1 (14.3%) |
| **CIS** | 1 (14.3%) |
| **T2** | 4 (57.1%) |
| **T4** | 2 (28.6%) |

*Supplemental Table 1D. Demographic overview of non-recurrent* patient samples.

|  | **Non-Recurrent Patients** |
| --- | --- |
| **Total Patients** | 35 |
| **Age Mean** | 63.5 |
| **Age Range** | 32-81 |
| **Males** | 25 (71.4%) |
| **Females** | 10 (28.6%) |
| **Former Smokers** | 7 (20%) |
| **Current Smokers** | 11 (31.4%) |
| **Never Smokers** | 17 (48.6%) |
| **CIS** | 3 (8.6%) |
| **T1** | 10 (28.6%) |
| **T2** | 6 (17.1%) |
| **T3** | 4 (11.4%) |
| **T4** | 9 (25.7%) |
| **Dysplasia** | 1 (2.9%) |
| **Unknown** | 2 (5.7%) |

**Supplemental Table 2. List of TaqMan miRNA primers included on each step of the classifier development.**

| **TaqMan microRNA Assay ID** | **miRNA** | **14 miRNA Panel** | **6 miRNA Panel** | **2 miRNA Panel** | **Note** |
| --- | --- | --- | --- | --- | --- |
| 000400 | hsa-miR-23b | Yes | Yes | Yes | Endogenous Control |
| 000449 | hsa-miR-125b | Yes | Yes | Yes |  |
| 002260 | hsa-miR-342-3p | Yes | Yes | Yes |  |
| 000399 | hsa-miR-23a | Yes | Yes |  |  |
| 000509 | hsa-miR-205 | Yes | Yes |  |  |
| 002135 | hsa-miR-33a | Yes | Yes |  |  |
| 002278 | hsa-miR-145 | Yes | Yes |  |  |
| 000387 | hsa-miR-10a | Yes |  |  |  |
| 000464 | hsa-miR-142-3p | Yes |  |  |  |
| 001544 | hsa-miR-550 | Yes |  |  |  |
| 001589 | hsa-miR-616 | Yes |  |  |  |
| 001998 | hsa-miR-769-5p | Yes |  |  |  |
| 002165 | hsa-miR-29b-1* | Yes |  |  |  |
| 002300 | hsa-miR-200c | Yes |  |  |  |
| 002446 | hsa-miR-28-3p | Yes |  |  |  |

**Supplemental Table 3: LASSO selection of miRNAs for analysis of SYBR qRT-PCR with the inclusion of linearized CT values as well as their squares and roots.**

| **Order** | **Action** | **miRNA** | **Exponent** |
| --- | --- | --- | --- |
| 1 | Add | hsa-miR-23a-3p | 1 |
| 2 | Add | hsa-miR-342-3p | 1 |
| 3 | Add | hsa-miR-145-5p | 2 |
| 4 | Add | hsa-miR-205-5p | 1 |
| 5 | Add | hsa-miR-142-3p | 1 |
| 6 | Add | hsa-miR-125b-5p | 1 |
| 7 | Add | hsa-miR-33a-5p | 1 |
| 8 | Add | hsa-miR-200c-3p | 1 |
| 9 | Add | hsa-miR-29b-1* | 1 |
| 10 | Add | hsa-miR-28-3p | 4 |
| 11 | Add | hsa-miR-550a | 1 |
| 12 | Add | hsa-miR-616 | 1 |
| 13 | Add | hsa-miR-769-5p | 2 |
| 14 | Add | hsa-miR-10a-5p | 1 |

**Supplemental Table 4: miRNAs selected after 9 LASSO actions on TaqMan training set data.**

| **miRNA** | **Exponent** |
| --- | --- |
| hsa-miR-125b-5p | 0 |
| hsa-miR-342-3p | 0 |
| hsa-miR-23a-3p | 0 |
| hsa-miR-205-5p | 0 |
| hsa-miR-145-5p | 2 |
| hsa-miR-33a-5p | 0 |
| hsa-miR-145-5p | 0 |
