# Supplementary material for: Identification of a serum-based microRNA signature that detects recurrent oral squamous cell carcinoma before it is clinically evident
Source: Br J Cancer. 2023 Oct 5;129(11):1810–7. doi: 10.1038/s41416-023-02405-9 (PMC10667517; doi:10.1038/s41416-023-02405-9)
Supplement: Supplementary file 2 — Supplemental Figures [file 41416_2023_2405_MOESM2_ESM.pdf]

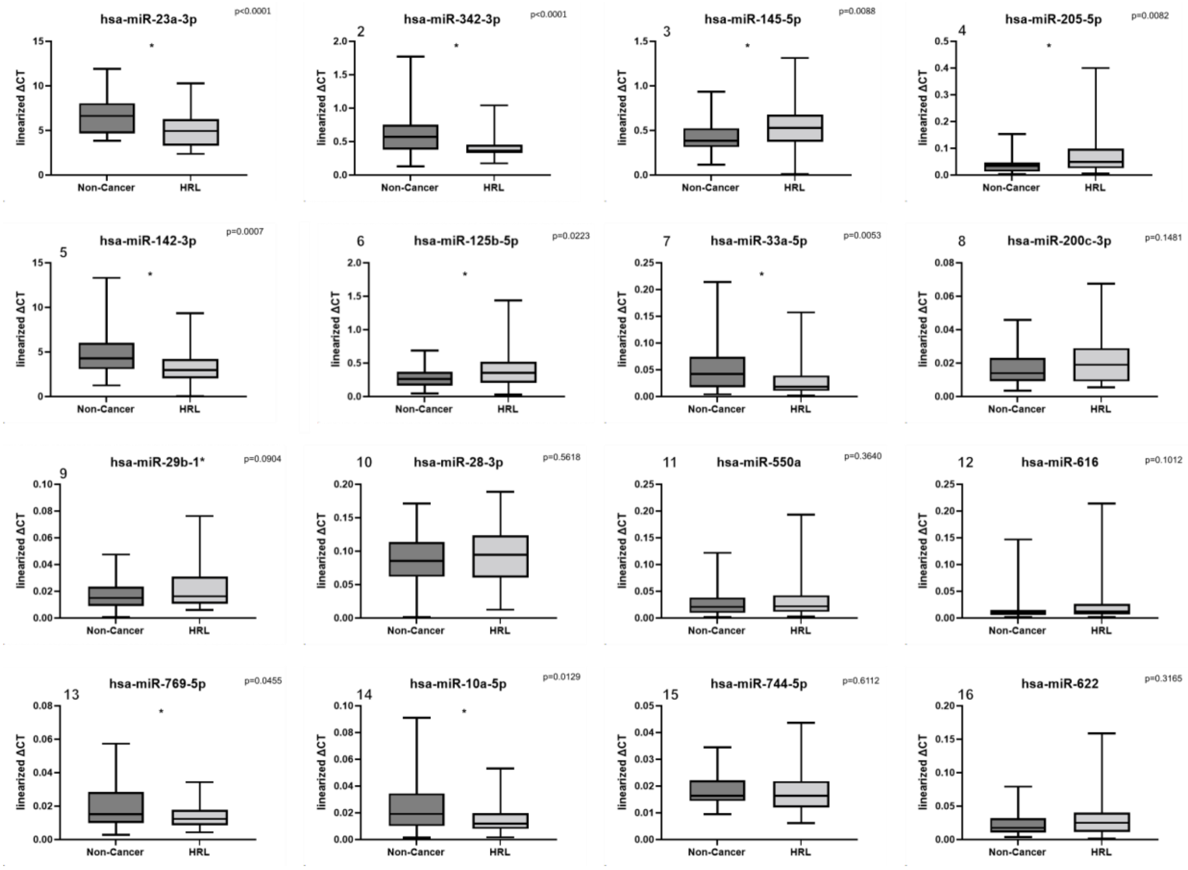

Supplemental Figure 1. Box plots indicating linearized delta Ct values for the top contributing miRNA in the LASSO analysis using the SYBR qPCR platform. P-values were calculated using the Mann-Whitney U test. An asterisk denotes statistical significance.

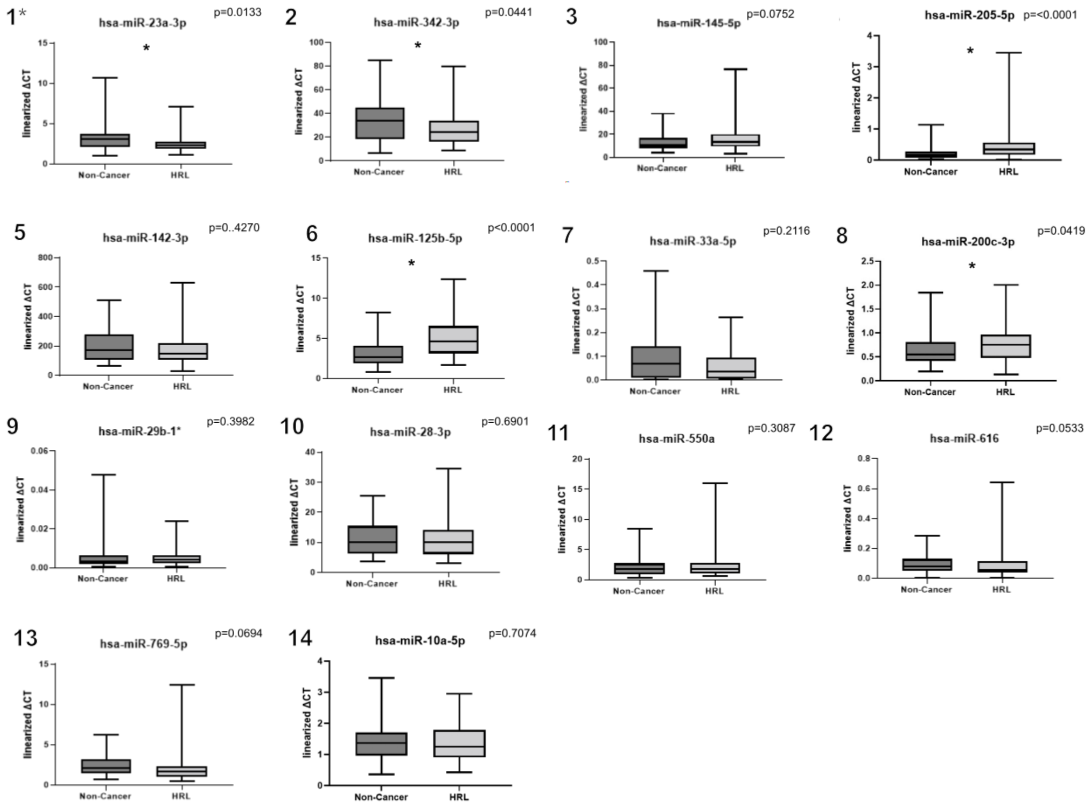

Supplemental Figure 2. Box plots indicating linearized delta Ct values for top scoring microRNA using the TaqMan qPCR platform. P-values were calculated using the Mann-Whitney U test. An asterisk denotes statistical significance.

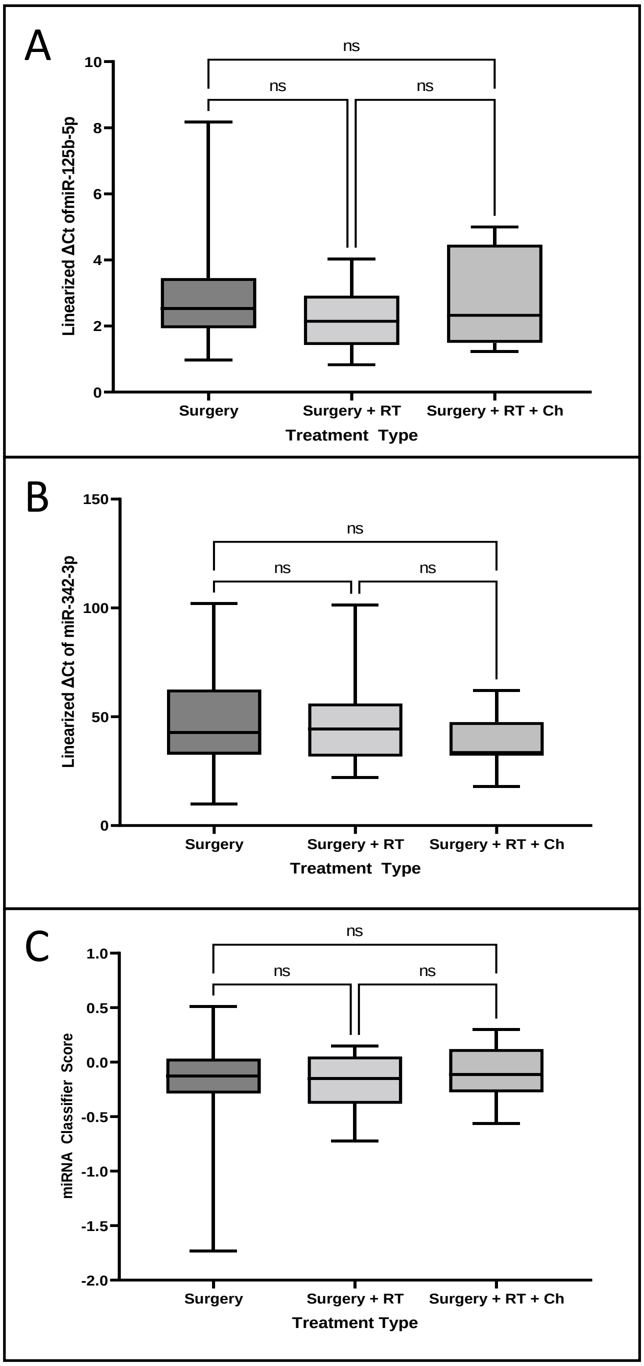

Supplemental Figure 3. Effect of treatment type on linearized ΔCT values of (A) miR-125b-5p, (B) miR-342-3p alone, and (C) combined miR-342-3p and miR-125b-5p.

ΔCT = −ΔCT = Ct(reference miR-23b-5p) – Ct(miR of interest).

(Surgery n=91, Surgery + RT n=19, Surgery + RT + Chemo n=10). Significance determined by Kruskal-Wallis test.
